# Narcotic analgesic utilization amongst injured workers: using concept mapping to understand current issues from the perspectives of physicians and pharmacists

**DOI:** 10.1186/1472-6963-11-280

**Published:** 2011-10-20

**Authors:** Janet A Parsons, Muhammad Mamdani, Onil Bhattacharyya, Claire Marie Fortin, Magda Melo, Christina Salmon, Stavroula R Raptis, Donna Bain, Patricia O'Campo

**Affiliations:** 1 Applied Health Research Centre, Li Ka Shing Knowledge Institute, St. Michael's Hospital, 30 Bond St., Toronto, Ontario, M5B 1W8, Canada; 2 Dept. of Physical Therapy, University of Toronto, Suite 160, 500 University Ave., Toronto, Ontario, M5G 1V7, Canada; 3 Dept. of Health Policy Management & Evaluation, University of Toronto, Health Sciences Building, Suite 425, 155 College St., Toronto, Ontario, M5T 3M6, Canada; 4 Leslie Dan Faculty of Pharmacy, University of Toronto, 144 College St., Toronto, Ontario, M5S 3M2, Canada; 5 Keenan Research Centre, Li Ka Shing Knowledge Institute, St. Michael's Hospital, 30 Bond St., Toronto, Ontario, M5B 1W8, Canada; 6 Dept. of Family & Community Medicine, University of Toronto, 5th Floor, 256 McCaul St., Toronto, Ontario, M5T 1W7, Canada; 7 Canadian Institute for Health Information, Suite 300, 4110 Yonge St., Toronto, Ontario, M2P 2B7, Canada; 8 Centre for Research on Inner City Health, Li Ka Shing Knowledge Institute, St. Michael's Hospital, 30 Bond St., Toronto, Ontario, M5B 1W8, Canada; 9 Health Services Division, Workplace Safety and Insurance Board of Ontario, 200 Front St. West, Toronto, Ontario, M5V 3J1, Canada; 10 Dalla Lana School of Public Health, University of Toronto, Health Sciences Building, 6th Floor, 155 College St., Toronto, Ontario, M5T 3M6, Canada

## Abstract

**Background:**

Work-related injuries result in considerable morbidity, as well as social and economic costs. Pain associated with these injuries is a complex, contested topic, and narcotic analgesics (NA) remain important treatment options. Factors contributing to NA utilization patterns are poorly understood. This qualitative study sought to characterize the factors contributing to NA utilization amongst injured workers from the perspectives of physicians and pharmacists.

**Methods:**

The study employed concept mapping methodology, a structured process yielding a conceptual framework of participants' views on a particular topic. A visual display of the ideas/concepts generated is produced. Eligible physicians and pharmacists (n = 22) serving injured workers in the province of Ontario (Canada) were recruited via purposive sampling, and participated in concept mapping activities (consisting of brainstorming, sorting, rating, and map exploration). Participants identified factors influencing NA utilization, and sorted these factors into categories (clusters). Next, they rated the factors on two scales: 'strength of influence on NA over-utilization' and 'amenability to intervention'. During follow-up focus groups, participants refined the maps and discussed the findings and their implications.

**Results:**

82 factors were sorted into 7 clusters: *addiction risks, psychosocial issues, social/work environment factors, systemic-third party factors, pharmacy-related factors, treatment problems*, and *physician factors*. These clusters were grouped into 2 overarching categories/regions on the map: patient-level factors, and healthcare/compensation system-level factors. Participants rated NA *over-utilization *as most influenced by patient-level factors, while system-level factors were rated as most amenable to intervention. One system-level cluster was rated highly on both scales (*treatment problems *- e.g. poor continuity of care, poor interprofessional communication, lack of education/support for physicians regarding pain management, unavailability of multidisciplinary team-based care, prolonged wait times to see specialists).

**Conclusions:**

Participants depicted factors driving NA utilization among injured workers as complex. Patient-level factors were perceived as most influential on over-utilization, while system-level factors were considered most amenable to intervention. This has implications for intervention design, suggesting that systemic/structural factors should be taken into account in order to address this important health issue.

## Background

It is estimated that over 100 million occupational injuries occur each year worldwide [1]. Although estimates vary considerably, most industrialized nations report high rates of work-related injury [2-5]. The social and economic costs associated with such injuries are substantial. In 2006, American employers spent $87.6 billion on workers' compensation, although this does not reflect personal and social costs [6]. In Europe, approximately 150 million work days are lost annually to work-related injuries [7]. Work-related injuries are frequently painful, with their management complicated by the subjective nature of pain, uncertainty around the care of persisting, ill-defined, or complex conditions, and difficulties experienced by clinicians dealing with both injured workers and employers [8]. While numerous pharmacologic and non-pharmacologic treatments for pain exist, opioid analgesics remain important options for relieving pain [9]. The advent of new agents has been accompanied by increased prescription of narcotic analgesics (NA) in industrialized nations, raising concerns about potential abuse [9-11]. These concerns have been fueled by recent media attention, but empirical studies have yielded mixed results - some indicating increasing adverse outcomes and evidence of abuse, others finding no change in the overall pattern [9-13].

Pain associated with work-related injury is a highly complex and contested topic. Review of the return-to-work (RTW) literature reveals little agreement regarding how to define and operationalise outcomes, with pain frequently dismissed as 'subjective' [14]. Pain management in injured workers is likewise plagued by controversy, with reports of increasing trends toward early NA prescription for certain conditions (e.g. occupational low back pain) despite guidelines recommending NA use only in rare cases [15-17].

Recent interest in social scientific approaches to RTW stems from growing recognition that RTW is not purely a biophysical process, but rather the product of multiple influences (e.g. psychological, social, structural/environmental) [14,18-21] . Understanding the RTW process from the perspectives of injured workers and health care practitioners is important when interpreting observed behaviours and outcomes. Prior studies eliciting workers' and employers' perspectives on RTW have revealed important barriers to the uptake of policies and practices [22,23], but the issue of NA utilization in the context of work-related injury has not been addressed specifically using qualitative research methods.

A few qualitative studies focused on NA utilization specifically (outside RTW contexts) have been conducted. Butler and colleagues used concept mapping methodology to elicit patients' and practitioners' perspectives on drug-taking behaviours, but did not explore factors external to the patient [24-26]. Few studies have sought the perspectives of practitioners regarding NA prescription practices. A Norwegian study of prescribers showed a general tendency to defer responsibility to the previous physician, and to patient autonomy [27]. However, these authors did not address work-related injury.

Any attempt to improve quality of care in the RTW arena (at the system, practitioner or worker level) must first attempt to understand the factors driving current practice patterns. There were two primary aims of this study: 1) to understand the factors contributing to NA utilization amongst injured workers from the perspectives of physicians and pharmacists; and 2) to explore physicians' and pharmacists' perspectives of how amenable these proposed factors are to intervention. A secondary aim was to compare perceptions between physicians and pharmacists, as it was possible that the two professions might view the issue differently.

## Methods

### Study design and participants

Qualitative methods are well-suited to studying complex phenomena, including the contexts in which people live, work and receive care [20,21]. Healthcare organization, contextual factors, and the experiences and perspectives of key stakeholders shaping practice behaviours - all may be studied using qualitative methods. We conducted a qualitative study using concept mapping (CM) methodology. CM is "a structured process, focused on a topic or construct of interest, entailing input from ... participants, that produces an interpretable pictorial view (concept map) of their ideas .... and how these are interrelated" [28]. Although primarily qualitative in nature, CM has features of both qualitative and quantitative approaches, entailing qualitative data collection coupled with quantitative analytic techniques which are used to create a visual display of how participants conceptualize the topic. CM is comprised of three activities: 1) group-based brainstorming, 2) individual web-based sorting and rating, and 3) group exploration of the maps. Data gathering activities are completed so that each participant's viewpoint is represented while incorporating group consensus (described below). This method was proposed because it considers a wide array of factors and organizes them into a coherent framework.

This was a collaborative project between St. Michael's Hospital and the Workplace Safety and Insurance Board of Ontario (WSIB). Data collection occurred from March to September 2008, at St. Michael's Hospital (Toronto, Ontario, Canada). The research protocol was approved by the hospital's Research Ethics Board.

WSIB identified eligible participants from an administrative database of physicians and pharmacies serving injured workers. An invitation letter was sent to potential participants informing them about the study. Inclusion criteria were: physician or pharmacist licensed to practice in Ontario; recent experience prescribing or dispensing NA; registered as serving WSIB claimants; and practicing within the greater Toronto area. Physician and pharmacist RTW experts at the compensation board (with relevant practice experience) were also invited to participate. In keeping with qualitative methodology, purposive sampling was conducted to promote participation of those with varied levels of experience in clinical practice.

### Data collection and analytic procedures

While detailed descriptions of the CM method are documented elsewhere [28-30], we outline each step briefly below, including relevant analytic activities.

Brainstorming sessions (1.5 - 2 hours) were conducted with three separate groups: community-based pharmacists, community-based physicians, and compensation board expert clinicians (physicians and pharmacists). The rationale for holding separate groups for community-based physicians and pharmacists was to decrease the possibility of social desirability bias, whereby one set of professionals might not feel comfortable sharing their views with another (potential power differential). There were insufficient numbers of pharmacists among the compensation board experts to hold a separate group with these individuals. Participants were asked to complete the following sentence: "A factor which influences NA utilization amongst patients experiencing work-related injuries/illnesses is ..." Participants were encouraged to generate as many statements as possible that would complete this sentence. A definition of 'NA' was provided to ensure consistency within and across groups and read as follows: "*Narcotic analgesics *(NA) are pain relievers that work on the central nervous system (CNS) to alter the perception of pain. Examples are: codeine, fentanyl, hydrocodone, hydromorphone, meperidine, methadone, morphine, & oxycodone. For the purposes of this study, we are interested in narcotic analgesics as prescribed for the treatment of chronic non-malignant pain in patients who have experienced work-related injuries or illnesses." Item lists from all groups were merged (duplicates eliminated and like items consolidated), to yield a master list capturing all unique ideas generated.

Sorting and rating of items was then conducted online individually using Concept Systems (CS) Global software (version 4) [31]. Participants were first asked to sort the items into groups that "made sense to them" and to label them accordingly to reflect the items within. Next, participants rated each factor according to their perceptions of:

a) how strongly it influences NA *over*-utilization (rated as 1 = not at all (does not influence NA over-utilization) to 5 = very strongly influences NA over-utilization)

b) how amenable it is to intervention (where 1 = not at all amenable and 5 = extremely amenable)

Upon completion of sorting and rating, data were exported into the CS Core system, which is necessary in order to conduct multidimensional scaling and hierarchical cluster analysis [30]. Information about the distance of each item to all other items was produced to illustrate clusters of items, representing conceptual domains. All participants' data were used to produce a single aggregate map representing the collective viewpoint of participants.

The aggregate map was shared with participants in follow-up focus groups for confirmation and further discussion and refinement [30]. Fifteen participants returned for this activity, confirming the appropriate number of clusters, the positioning of items within clusters, and refining the cluster labels. A 7-cluster solution was confirmed and consensus reached. Based on feedback from these focus groups, the final aggregate map was produced, with a stress value of 0.27 (well within the acceptable range reported in the CM literature, indicating excellent goodness-of-fit for the resulting model) [30]. Finally, pattern matches and go-zones were employed to look at relative agreement on the ratings between groups (e.g. between physicians and pharmacists, those with lower versus those with higher levels of practice experience, and between compensation board experts and community-based practitioners) [30].

## Results

Pharmacists were contacted for participation via 22 local pharmacies identified from the WSIB dispensing database as high-volume dispensers of NA to injured workers. Fourteen declined participation, most (n = 12) citing staff shortages, long work hours and travel distance to attend the group sessions as the primary reasons for refusal. Eight community-based pharmacists took part in the study. Thirty-eight eligible local community-based physicians were identified and contacted (first by letter then by phone). Of these, 4 could not be reached because of incomplete/incorrect contact information, 10 did not call back despite repeated attempts, and 15 declined to participate outright. Nine community-based physicians expressed interest and willingness to participate in the study, but only 6 were ultimately available to attend the group sessions. Compensation board expert clinicians (physicians and pharmacists) were identified from within the consultant staff at the WSIB and invited to participate in the brainstorming sessions. Eight practitioners participated. Compensation board participants were also community-based practitioners.

A total of 22 participants attended the brainstorming groups (13 physicians, 9 pharmacists). Ten were men and 12 were women, with a mean age of 46 years (range = 29 - 64). Mean number of years practicing their designated profession was 18 (range = 2 - 40 years). Eight compensation board expert clinicians (mean practice experience = 14.9 years, range = 4 - 30 years), 6 community-based physicians (mean experience = 18.3 years, range = 2 - 36 years) and 8 community-based pharmacists (mean experience = 21.3 years, range = 7 - 40 years) participated in the brainstorming groups. More participants took part in brainstorming with 4 fewer of the original 22 participants performing sorting and rating (n = 18; 10 physicians, 8 pharmacists), which is common and acceptable practice in CM projects [30]. These 4 persons were unavailable during the scheduled period for this activity.

### Relevant factors and concept map

Additional File 1 presents the master list of 82 items generated by participants (grouped by cluster label). These factors ranged from those related to patients/workers themselves (including psychosocial context), medication, work environment, healthcare and compensation systems, to prescriber and dispenser factors.

**Figure 1:**
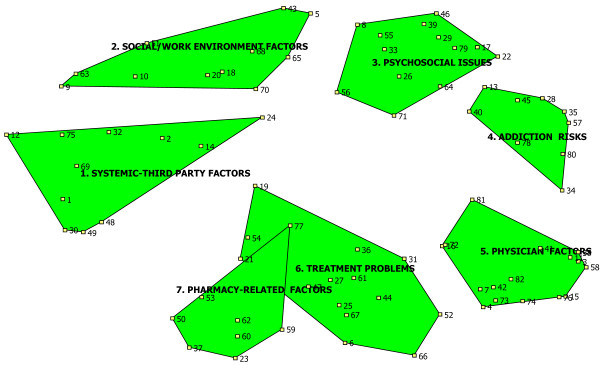
**Cluster Map**. Items in each cluster are denoted by numbers which correspond to the item numbers in Additional file 1. The numbers are not ordered or ranked, but simply correspond to the item they denote.

Participant sorting activities yielded point maps and cluster maps. The final 7-cluster map is presented in Figure 1. Items in each cluster are denoted by numbers which correspond to the item numbers in the table in Additional File 1. The numbers are not ordered or ranked, but simply correspond to the item they denote. Items that appear closer on the map are perceived by participants to be more closely related to each other. Figure 1 represents a unique conceptualization of factors perceived to be influencing NA utilization amongst injured workers. The clusters and their associated factors can be summarized as follows: (1) *systemic-third party factors *(including relationship between workers and compensation board, claims issues, lack of workplace accommodations, etc.), (2) *social/work environment factors *(e.g. stigma of being on compensation, lack of family and other supports, job description, unsupportive attitudes of employers, etc.);

(3) *psychosocial issues *(e.g. motivation to RTW, socioeconomic status, experience of disability, cultural norms and expectations); (4) *addiction risks *(e.g. presence of comorbidities, previous history of addiction, poor patient-physician relationship, noncompliance); (5) *physician factors *(e.g. clinician's expectations around pain management, physician's attitude/belief in patient's pain, and pressures on physicians to see large volume of patients in fee-for-service healthcare system); (6) *treatment problems *(including lack of continuity of care, long wait times to see specialists, clinician lack of awareness of non-NA options, poor availability of multidisciplinary team-based care, poor physician education in pain management); and (7) *pharmacy-related factors *(e.g. the addictive nature of the NA initially prescribed, use of long-acting rather than short-acting NAs, poor availability of effective alternative medications, barriers to pharmacists educating patients, availability of online drug plans/prescription databases). Additional File 1 provides a detailed consideration of all 82 factors grouped by cluster.

The reader is reminded that this conceptualization incorporates the *perspectives *of physicians and pharmacists experienced in working with this population. Each cluster represents a conceptual domain, with the 7 cluster labels developed by the participants themselves, using their own words and through a process of consensus. The 7 categories generated can be seen as clustering into two over-arching regions on the map, with the 3 clusters in the upper half comprising one region and the bottom 4 clusters comprising another:

1) The upper region (Social/Work Environment Factors, Psychosocial Issues and Addiction Risks) is composed of *factors related to the injured worker and the context in which they live, work and receive care*.

2) The lower region (Systemic-Third Party Factors, Pharmacy-Related Factors, Treatment Problems and Physician Factors) deals with *system-level factors *related to the healthcare and compensation systems.

### Rating maps

**Figure 2:**
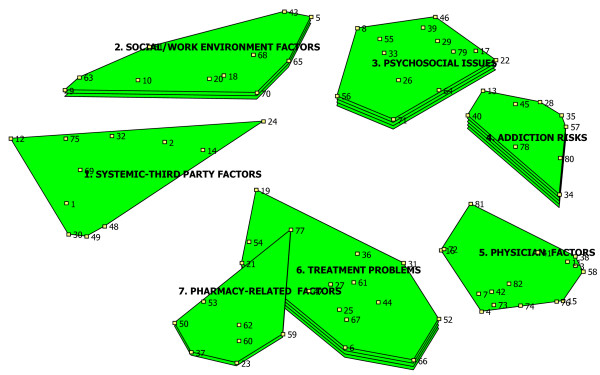
**Ratings: Strength of Influence on NA over-utilization**.

**Figure 3:**
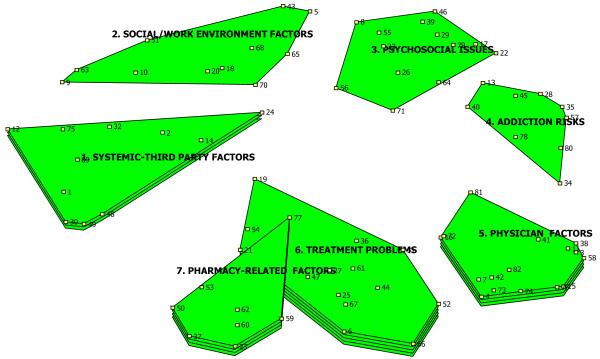
**Ratings: Amenability to Intervention**.

Clusters with more 'layers' are more highly rated by participants, while those with no layers (flat clusters) are seen as less important (Figures 2 and 3).

#### Factors influencing NA over-utilization amongst injured workers

Figure 2 demonstrates that several clusters were seen as influencing NA over-utilization. The cluster 'Addiction Risks' was rated most influential (4 layers), while the clusters 'Treatment Problems' and 'Psychosocial Issues' were next most influential (3 layers each). By contrast, 'Systemic-Third Party Factors' and 'Physician Factors' were seen as having less influence on NA over-utilization (0 layers each). Table 1 provides more detailed information concerning individual items contributing to the ratings among two highly-rated clusters ('Addiction Risks', 'Treatment Problems').

**Table 1:** Factors (items) influencing cluster ratings for NA over-utilization

Cluster	Influential items (factors) within cluster	Item #
***Addiction Risks***		

	patient's history of NA use	**28**

	patient's history of addiction	**45**

	presence of comorbid conditions in patient (including psychological/mental)	**40**

	patient's request for NA	**35**

***Treatment Problems ***		

	poor or absent protocols for weaning patients off NA	**27**

	lack of education/support for physicians in pain management (i.e. lack of guidelines)	**52**

	unavailability of multidisciplinary team-based care	**44**

	inability to get non-pharmacological treatments (e.g. physiotherapy, acupuncture, massage therapy)	**54**

	lack of continuity of care	**36**

	lack of resources in assisting patients with NA addiction/withdrawal	**21**

#### Factors amenable to intervention

Figure 3 is a cluster rating map depicting participants' perspectives concerning the factors' relative amenability to intervention (to optimize NA utilization). Factors within the cluster 'Treatment Problems' were seen as most amenable to intervention (4 layers), while the clusters 'Physician Factors' and 'Pharmacy-Related Factors' (3 layers each) were seen as next-most-amenable to intervention. By contrast, the clusters 'Addiction Risks', 'Psychosocial Issues' and 'Work/Social Environment Factors' were *not *seen as amenable to intervention by participants. Table 2 provides more detailed information concerning individual items contributing to the ratings among the three most highly rated clusters ('Treatment Problems', 'Physician Factors' and 'Pharmacy-related Factors').

**Table 2:** Factors (items) influencing cluster ratings for amenability to intervention

Cluster	Influential items (factors) within cluster	Item #
***Treatment Problems ***		

	prolonged time to accurate diagnosis	**31**

	lack of continuity of care	**36**

	poor interprofessional communication	**66**

	lack of education/support for physicians in pain management (i.e. lack of guidelines)	**52**

	unavailability of multidisciplinary team-based care	**44**

	inability to get non-pharmacological treatments (e.g. physiotherapy, acupuncture, massage therapy)	**54**

	lack of resources in assisting patients with NA addiction/withdrawal	**21**

	pharmacist and physician lack of knowledge of non-NA options	**25**

***Physician Factors***		

	clinician's expectations around pain management (e.g. goal of 'zero pain')	**38**

	(lack) of a contract between physicians and patients (narcotic contract)	**76**

***Pharmacy-related Factors***		

	poor availability of effective alternative medications (including non-narcotic medications)	**53**

	use of long-acting rather than short-acting NAs	**59**

	availability of drug plan online, so pharmacist is aware of number of prescriptions, frequency of refills, available substitutions	**37**

	no upper limit of NA dosage	**60**

### Comparing the two rating maps

If we subdivide the maps into 2 over-arching regions (upper comprised of 3 patient-related clusters, lower of 4 system-level clusters) the rating distributions indicate that participants perceived *patient-related factors to be driving NA over-utilization*. However, the factors perceived to be *most amenable to intervention are system-level factors *(i.e. those operating external to injured workers). Only one system-level cluster ('Treatment Problems') was rated highly on both scales. Otherwise, there was little overlap in terms of what was considered most influential to NA over-utilization and relative amenability to intervention.

**Figure 4:**
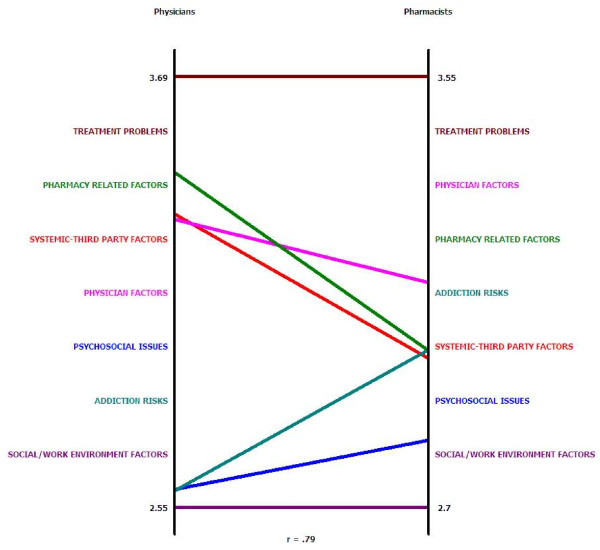
**Pattern Match: Comparing Physician and Pharmacist Cluster Ratings - Amenability to Intervention**.

Finally, we generated pattern matches and go-zones to examine the extent to which physicians and pharmacists agreed on cluster ratings [30]. Pattern matches yield simple 'ladder graphs', with the two vertical axes representing the mean ratings for each cluster [26]. Figure 4 depicts relative inter-disciplinary agreement regarding cluster ratings on amenability to intervention. Physician ratings appear on the left and pharmacist ratings on the right. Each horizontal line between the vertical axes represents a cluster on the concept map. A correlation value of 0.79 at the bottom of the graph indicates that there is relatively high agreement between the disciplines [30]. The horizontal lines for 'Treatment Problems' at the top (highly rated by both disciplines) and at the bottom for 'Social/Work Environment Factors' (rated low by both disciplines) indicate a high degree of agreement on these particular clusters. The differences evident in the middle of the graph likely reflect differences based on experiences and role of each discipline in managing pain in injured workers.

**Figure 5:**
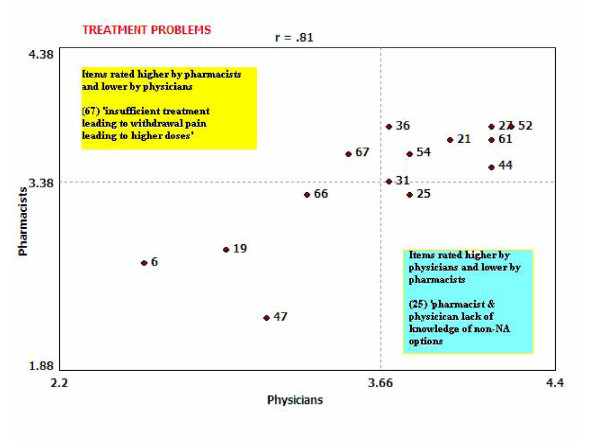
**Go-Zone: Comparing Physician and Pharmacist Item Ratings of 'Treatment Problems' Cluster - Amenability to Intervention**.

Go-zones are XY graphs of ratings divided into quadrants based on the mean for each variable [30]. Figure 5 depicts the relative agreement between physicians and pharmacists regarding their ratings of each factor's amenability to intervention within the 'Treatment Problems' cluster specifically. This graph demonstrates a high degree of agreement on the ratings between both groups (r = 0.81) [30]. The upper left quadrant of the graph includes items rated higher by pharmacists and lower by physicians. Only one item sits in this quadrant (67, 'insufficient treatment, leading to withdrawal pain, leading to higher doses'). The right lower quadrant includes items rated higher by physicians and lower by pharmacists. Again, only one item sits in this quadrant (25, 'pharmacist and physician lack of knowledge of non-NA options'). Those items in the left lower quadrant indicate items that were rated lower by both pharmacists and physicians, while those in the upper right quadrant indicate items that were rated highly by both disciplines - again indicating a high degree of agreement on most item ratings in this cluster. Appendix 1 provides a brief description of additional comparisons conducted.

## Discussion

This study represents an original contribution by producing a conceptual map of factors influencing NA utilization amongst injured workers, drawing specifically on the perspectives of physicians and pharmacists. Participants portrayed a complex and multifaceted phenomenon, depicting injured workers within their personal and social contexts, and identifying numerous factors operating at multiple levels. This is the first study to characterize the topic in a comprehensive manner, and to attempt to interrelate the contributing factors within a coherent framework.

Surprisingly, participants saw system-level factors as more amenable to intervention, despite their perceptions that patient-level factors are most influential to NA *over*-utilization in this population. Numerous quantitative studies of NA usage have been conducted (some in the context of RTW), and the majority focus on identifying individual factors/characteristics that put patients at risk for abuse (e.g. age, psychiatric comorbities, history of substance abuse) [15-17,32,33]. Russell and colleagues (2005) conducted a qualitative study of family practitioners related to managing RTW patients generally (not focused on NA) which revealed that physicians viewed 'external influences' (compensation boards, employers, etc.) with suspicion [8]. In contrast, our participants indicated that they would welcome further guidance in managing workers from a variety of sources (including compensation boards). Interestingly, a large scale quantitative study by Shaw and colleagues (2005) looking at RTW reported that RTW and shorter duration of disability were predicted by employment factors rather than individual patient history and physical examination [34].

Participants identified only one cluster of system-level factors ('Treatment Problems') as both strongly influencing NA overuse and highly amenable to intervention. This suggests that addressing the factors within this cluster is where interventions hold greatest promise of impacting NA utilization. This was surprising because to date, most interventional studies addressing NA overuse have focused on individual behaviour change (patient-level) [35-41]. Our clinician participants instead indicated that there were changes at the system-delivery level that could be implemented to improve NA utilization amongst injured workers. This demonstrates the importance of employing *both *rating maps to understand the problem and its potential solutions. If we relied solely on Figure 2 (perceived drivers of over-utilization), we might erroneously conclude that interventions should be targeted primarily at the level of patient/worker behaviour change. Instead, participants identified practice- and system-level factors as more amenable to intervention. Participants rated one cluster ('Treatment problems') highly on both rating scales (important influencers on overutilization *and *most amenable to change), suggesting that this might be the set of factors to which interventions should be targeted to improve outcomes. Tables 1 and 2 indicate that five factors specifically were ranked as particularly important to NA overutilization as well as being amenable to intervention. These were: 'lack of education/support for physicians in pain management' (52), 'unavailability of multidisciplinary team-based care'(44), 'inability to get non-pharmacologic treatments' (54), 'lack of continuity of care' (36), and 'lack of resources in assisting patients with NA addition/withdrawal'(21). This suggests that optimizing collaboration between physicians, pharmacists, clinical specialists, compensation experts, and injured workers may be most important to improving worker well-being and reducing the associated costs of time off work. That being said, the care of workers with chronic pain is complex and multifaceted approaches will be needed, with implementation of strategies targeting providers and treatment, coupled with an exploration of novel approaches to address patient-level factors. Intervention studies would have to be conducted to determine whether focusing on these factors translates into reductions in NA use.

Given that participants emphasized the lack of education/support for physicians in managing pain in injured workers, an intervention such as academic detailing might be an appropriate initial step to improving practice [42]. Such approaches incorporate face-to-face interactions with trusted expert clinicians, and are thought to be more successful than traditional guideline dissemination approaches to changing practice [43]. Participants suggested a number of avenues for improving interprofessional communication between physicians and pharmacists (e.g. through an electronic prescription monitoring system) [44], and an enhanced role for compensation boards for communicating existing community-based resources for accessing specialty services and non-pharmacologic care.

While there was considerable agreement between physicians and pharmacists regarding the cluster ratings, the minor differences identified on the pattern matches and go-zones are intriguing. These may be largely explained by the specific roles physicians and pharmacists play in the pain management of injured workers. In the pattern match, pharmacists ranked 'Addiction Risks' as more amenable to intervention than physicians. It may be that pharmacists are more aware than family physicians regarding strategies for managing addiction risks. Conversely, physicians ranked 'Systemic-Third Party Factors' as more amenable to intervention than pharmacists. This could be explained by the fact that physicians have more interaction with compensation boards (emphasized in the cluster 'Systemic-Third Party Factors') than do pharmacists, and so are likely more aware of opportunities for process redesign within that cluster. Interestingly, physicians ranked 'Pharmacy-related Factors' as more amenable to intervention than 'Physician-related Factors', while pharmacists were more optimistic that 'Physician-related Factors' could be improved than were physicians themselves.

While ours is the first study to address this topic using CM, others have used CM to explore opioid utilisation generally. Butler and colleagues conducted a series of CM studies (2004, 2006, 2007) as a preliminary step in developing patient self-report measures of prescription opioid abuse [24-26]. None of these studies evaluated injured workers, and Butler focused exclusively on *patient-level *drug-related behaviours. The purpose of using CM in our study was different, as we sought to catalogue a broad array of factors contributing to current NA utilization patterns (specifically in injured workers), and to organize them into a coherent framework. The study's results have confirmed that many factors beyond the injured worker are perceived to influence NA utilization. The participants in our study chose instead to emphasize the shortcomings of the healthcare system.

Two additional qualitative studies (neither employing CM nor focusing on work-related injury) have been conducted concerning NA prescription, one eliciting providers' perspectives, the other patients' [27,45]. Dybward and colleagues (1997) focused on NA prescription behaviours amongst Norwegian family doctors [27]. Like our participants, Norwegian practitioners depicted NA prescription as difficult work, but they emphasized patient-related factors (age, concomitant disease, autonomy) and a tendency to defer to the previous doctor to rationalize practice patterns [27]. Blake (2007) studied patients' perspectives of NA prescription for chronic pain, describing how patients balanced pain relief against concerns regarding side effects (and fear of addiction) [45]. The stigma of being perceived as addicted, the effects of pain on all aspects of their lives, and their ambivalence towards taking NA indicate that it is a complex process for patients as well [45]. The qualitative literature on RTW indicates that patients characterize the RTW journey as very complex and multi-faceted [14,22,23], far more so than is usually characterized by the clinicians interacting with them. The way RTW is characterized in the literature from practitioners' and epidemiologists' viewpoints is usually overly simplistic [18], with assumptions that workers simply are trying to return to their premorbid status. But we know that for persons experiencing major illness or injury, the journey back to work is shaped by many factors (e.g. nature of illness/injury, the type of treatment received, family structure and social supports, gender, age and life stage, education and socioeconomic status, the type of work engaged in (job demands), personal perspectives and societal discourses regarding disability, being on compensation, being unemployed, and shifting notions of the meaning of work in their lives) [14,23]. We also know that patients' and practitioners perspectives' often differ, whether concerning care received, health conditions treated, or expectations of recovery. For example, Kapoor and colleagues (2006) found that patient and physician expectations of RTW following occupational low back pain differed considerably [46]. A quantitative study by Mäntyselkä et al. (2001) comparing patients' and family physicians' ratings of patients' pain found considerable discordance between the groups, with physicians consistently ranking intensity lower than patients. The discordance became more pronounced as the severity of patients' ratings increased [47]. Future investigations using concept mapping methodology with injured workers is warranted.

### Limitations

This study has several limitations. First, we did not seek injured workers' perspectives, partly because of recent Canadian privacy regulations, and partly from concerns about not further stigmatizing this population. Instead, an exploratory study focusing on prescribers' and dispensers' views was seen as an appropriate *first *step to understanding NA utilization patterns. Second, these findings are based on practitioner *perceptions*. Nevertheless, perceptions inform behaviour and understanding, and as such offer valuable insights. Like all qualitative findings, ours are not meant to be generalisable at the population level. Rather we have produced a conceptual map highlighting linkages between factors from the perspectives of practitioners. While our participants practice in a particular jurisdiction (Ontario, Canada), the findings should be of interest to practitioners in other jurisdictions nationally and internationally. Concerns identified here - regarding interprofessional communication, coordination of care, availability of clinical practice guidelines, balancing pain relief against unwanted NA side effects - have been alluded to by international investigators [48-50].

## Conclusions

In summary, our study puts the context surrounding work-related injuries, NA utilization and clinician perspectives together, offering a conceptual framework for understanding factors contributing to this phenomenon. Not only have we identified factors perceived to influence NA over-utilization, but we have also identified factors perceived to be amenable to intervention. Surprisingly, while patient-related factors were seen as primary drivers of over-utilization, system-level factors were seen as most amenable to intervention. As such, these findings suggest areas for practice improvement.

## Competing interests

The authors declare that they have no competing interests.

## Authors' contributions

JAP was principal investigator for this project, and is responsible for its conceptualization, in addition to leading its design, data collection and analysis. She also had primary responsibility for drafting the manuscript. MM participated in the study's conceptualization and design, and contributed to the planning, content and format of the manuscript. OB contributed to the study design, data analysis and drafting the manuscript. CMF contributed content expertise related to workplace injury, as well as assistance with data interpretation. She also assisted with manuscript preparation. MM (author 5) assisted with the study design and preparation, coordination, as well as the data collection and analysis. She also contributed manuscript revisions. CS possesses expertise in concept mapping methodology and contributed to the design and implementation of the study, as well as participating in data collection and analysis. She also assisted with revisions to the manuscript. SRR assisted with the study design and coordination and contributed significantly to the data collection, analysis and interpretation of the findings. She also contributed to manuscript preparation. DB is an expert in work-related injury and worker compensation policy. She contributed to the conceptualization of the study and facilitated data acquisition. She also contributed to manuscript preparation. PO is an expert in concept mapping methodology and contributed significantly to the study's design, as well as the interpretation of the findings. She also contributed to manuscript preparation. All authors read and approved the final manuscript

## Appendices

### Appendix 1: Additional comparisons

While only comparisons between physicians and pharmacists are reported here, we also compared compensation board experts and community-based practitioners, as well as those with ≤ 10 years practice experience versus those with more than 10 years practice experience. Between-group ratings for the clusters on the pattern matches were highly correlated (r = 0.95) in both cases, indicating that compensation experts and community practitioners held very similar views, as did practitioners with varying years of practice experience.

## Pre-publication history

The pre-publication history for this paper can be accessed here:

http://www.biomedcentral.com/1472-6963/11/280/prepub

## Supplementary Material

Additional file 1 **Statement (Factor) List**. This table provides the full list of all 82 factors (items) generated by participants. The table also links each factor with its relevant cluster. *(Please see 'Additional files' uploaded with this article)*. Click here for file
