# Supplementary material for: Narcotic analgesic utilization amongst injured workers: using concept mapping to understand current issues from the perspectives of physicians and pharmacists
Source: BMC Health Serv Res. 2011 Oct 20;11:280. doi: 10.1186/1472-6963-11-280 (PMC3212981; doi:10.1186/1472-6963-11-280)
Supplement: Additional file 1 — Statement (Factor) List. This table provides the full list of all 82 factors (items) generated by participants. The table also links each factor with its relevant cluster. (Please see 'Additional files' uploaded with this article). [file 1472-6963-11-280-S1.DOC]

| # | **Label** | **Statements** |
| --- | --- | --- |
|  |  |  |
| 1 | **Systemic- Third Party Factors** | limited investigation and enforcement of suspected fraud due to lack of man-power (1) |
|  |  | smaller injuries morphing to greater pain (e.g., reflex sympathetic dystrophy, fibromyalgia) (2) |
|  |  | modified work insufficient if requires repetitive actions that still need NA (12) |
|  |  | medications that are costly to patient (14) |
|  |  | concerns about maintaining compensation/coverage (e.g., compensation board, drug plans) (24) |
|  |  | delayed involvement of medical consultant at compensation board on the claim (30) |
|  |  | pressures applied by compensation boards to patients (may be actual or perceived) (32) |
|  |  | lack of appropriate tests to properly examine work demands (48) |
|  |  | prolonged time for review of claim (by compensation board) (49) |
|  |  | problematic relationship between patients and compensation board/other insurers (75) |
|  |  | ease of reimbursement of compensation board prescription claims in recent years (100% is covered immediately) (69) |
| 2 | **Social/Work Environment Factors** | stigma associated with compensation claim (by workplace, by co-workers) (5) |
|  |  | severity of injury (e.g., traumatic vs non-traumatic) (9) |
|  |  | size of organization where patient is employed (10) |
|  |  | influence of media (in promoting public awareness, Internet as source of advice, providing alerts) (18) |
|  |  | ongoing or recurrent pain (20) |
|  |  | subjective nature of pain (43) |
|  |  | employer's unsupportive attitude towards patient's recovery (63) |
|  |  | social and environmental pressures to use/divert (family members sharing medications, income augmentation through diversion) (68) |
|  |  | poor support mechanisms from family, friends and co-workers (65) |
|  |  | job description (e.g., heavy physical demands, repetitive tasks, responsibilities) (51) |
|  |  | severity of pain (70) |
| 3 | **Psychosocial Issues** | family "budget" does not require them to work full-time (e.g., patient is not sole provider) (8) |
|  |  | NA gives patient perceived control over his/her condition (17) |
|  |  | patient's cultural norms and expectations around pain and its treatment (e.g., ethnicity, religious beliefs, local culture) (26) |
|  |  | patient's physical and emotional response to NA (29) |
|  |  | age of patient (influencing perspectives on NA and on work life stage) (22) |
|  |  | patient's outstanding legal issues (33) |
|  |  | patient's motivation to return to work (39) |
|  |  | stigma of being on a NA (46) |
|  |  | patient's education level and socio-economic status (55) |
|  |  | degree and duration of disability (56) |
|  |  | prescription seen by patient as affirmation of his/her suffering (64) |
|  |  | lack of injured worker knowledge of NA therapy and how injury is treated (71) |
|  |  | patient motivation for recovery (79) |
| 4 | **Addiction Risks** | patient non-compliance with health professionals' instructions (13) |
|  |  | patient's history of NA use (28) |
|  |  | poor relationship between patient and physician (34) |
|  |  | patient's request for NA (35) |
|  |  | presence of co-morbid conditions in patient (including history of psychological/mental health issues) (40) |
|  |  | patient's history of addiction (45) |
|  |  | patient gender (and perspective doctors have in prescribing to these groups) (57) |
|  |  | early development of dependency/addiction in patient (78) |
|  |  | condition that is visible ("justifiable pain") (80) |
| 5 | **Physician Factors** | physician's attitude and belief in patient's pain (3) |
|  |  | difficulty in interpreting information gathered from physician to physician (4) |
|  |  | pressure on physician to see large volume of patients in a fee-for-service system (7) |
|  |  | doctor's cultural perspective in prescribing (11) |
|  |  | physician's concerns about patient safety in work environment while on NA (i.e. patient ability to operate machinery) (15) |
|  |  | problematic diagnostic label or lack of diagnosis meeting rigorous criteria (16) |
|  |  | clinician's expectations around pain management (e.g., goal of "zero pain" in palliative care practise) (38) |
|  |  | overall tendency of physicians writing higher doses (42) |
|  |  | gender of physician (58) |
|  |  | lack of access to primary care (72) |
|  |  | physicians don't want to see compensation patients (73) |
|  |  | physician's lack of experience or past bad experience with NAs (74) |
|  |  | existence of a contract between physicians and patient (narcotic contract) (76) |
|  |  | intimidation by patient of health professionals (e.g., threat to call College of Physicians, physical intimidation) (81) |
|  |  | physician's reputation in community as NA-prescribing doctor (82) |
|  |  | prescribing to get rid of the patient (41) |
| 6 | **Treatment Problems** | pressure on pharmacist by physician/prescriber (6) |
|  |  | negative side effects of NA (19) |
|  |  | lack of resources in assisting patients with NA addiction/withdrawal (21) |
|  |  | pharmacist and physician lack of knowledge of non-NA options (25) |
|  |  | poor or absent protocols for weaning patients off of NA (27) |
|  |  | prolonged time to accurate diagnosis (31) |
|  |  | lack of continuity of care (36) |
|  |  | unavailability of multidisciplinary team-based care (44) |
|  |  | negative impact of drug company advertising on prescribing practices of physicians (47) |
|  |  | lack of education/support for physicians in pain management (i.e. lack of guidelines etc.) (52) |
|  |  | inability to get non-pharmacological treatments (e.g., physiotherapy, acupuncture, massage therapy) (54) |
|  |  | insufficient prescription, leading to withdrawal mediated pain, leading to higher doses (67) |
|  |  | long waiting times to see specialists (surgeons, pain clinics, etc.) (61) |
|  |  | poor inter-professional communication (e.g. between pharmacist and physician, with methadone clinic) (66) |
| 7 | **Pharmacy-related Factors** | barriers to pharmacist educating and communicating with patient (23) |
|  |  | availability of drug plan online so pharmacist is aware of number of prescriptions, frequency of refills, available substitutions (37) |
|  |  | poor availability of effective alternative medications (including non-narcotic medications) (53) |
|  |  | use of long-acting rather than short-acting NAs (59) |
|  |  | no upper limit of NA dosage (product monographs have no upper limit) (60) |
|  |  | addictive nature of NA prescribed initially (62) |
|  |  | poor access to pharmacists/pharmacy (77) |
|  |  | drug-drug interactions (with other prescribed medications) (50) |
|  |  |  |

N.B. The item numbers corresponding to the numbers appearing on the cluster map (Fig. 1) appear immediately after each factor (in brackets).
